# Gun-Cotton

**Published:** 1869-11

**Authors:** 


GUN-COTTON explodes when metallic sodium or metallic potassium is brought in contact with it. The amalgams of these metals do not produce the same effect. Finely divided arsenic requires percussion before it explodes cotton.—*Druggist's Circular.*

